# DiffusionFR: Species Recognition of Fish in Blurry Scenarios via Diffusion and Attention

**DOI:** 10.3390/ani14030499

**Published:** 2024-02-02

**Authors:** Guoying Wang, Bing Shi, Xiaomei Yi, Peng Wu, Linjun Kong, Lufeng Mo

**Affiliations:** 1 College of Mathematics and Computer Science, Zhejiang A&F University, Hangzhou 311300, China; wgy@zafu.edu.cn (G.W.); 2021611011034@stu.zafu.edu.cn (B.S.); yxm@zafu.edu.cn (X.Y.); wp@zafu.edu.cn (P.W.); 2 Office of Information Technology, Zhejiang University of Finance & Economics, Hangzhou 310018, China; 3 Information and Education Technology Center, Zhejiang A&F University, Hangzhou 311300, China

**Keywords:** blurry scenarios, fish recognition, deep learning, diffusion models

## Abstract

**Simple Summary:**

Blurry scenarios often affect the clarity of fish images, posing significant challenges to deep learning models in terms of the accurate recognition of fish species. A method based on deep learning with a diffusion model and an attention mechanism, DiffusionFR, is proposed herein to improve the accuracy of fish species recognition in blurry scenarios caused by light reflections and water ripple noise. Using a self-constructed dataset, BlurryFish, extensive experiments were conducted and the results showed that the proposed two-stage diffusion network model can restore the clarity of blurry fish images to some extent and the proposed learnable attention module is effective in improving the accuracy of fish species recognition.

**Abstract:**

Blurry scenarios, such as light reflections and water ripples, often affect the clarity and signal-to-noise ratio of fish images, posing significant challenges for traditional deep learning models in accurately recognizing fish species. Firstly, deep learning models rely on a large amount of labeled data. However, it is often difficult to label data in blurry scenarios. Secondly, existing deep learning models need to be more effective for the processing of bad, blurry, and otherwise inadequate images, which is an essential reason for their low recognition rate. A method based on the diffusion model and attention mechanism for fish image recognition in blurry scenarios, DiffusionFR, is proposed to solve these problems and improve the performance of species recognition of fish images in blurry scenarios. This paper presents the selection and application of this correcting technique. In the method, DiffusionFR, a two-stage diffusion network model, TSD, is designed to deblur bad, blurry, and otherwise inadequate fish scene pictures to restore clarity, and a learnable attention module, LAM, is intended to improve the accuracy of fish recognition. In addition, a new dataset of fish images in blurry scenarios, BlurryFish, was constructed and used to validate the effectiveness of DiffusionFR, combining bad, blurry, and otherwise inadequate images from the publicly available dataset Fish4Knowledge. The experimental results demonstrate that DiffusionFR achieves outstanding performance on various datasets. On the original dataset, DiffusionFR achieved the highest training accuracy of 97.55%, as well as a Top-1 accuracy test score of 92.02% and a Top-5 accuracy test score of 95.17%. Furthermore, on nine datasets with light reflection noise, the mean values of training accuracy reached a peak at 96.50%, while the mean values of the Top-1 accuracy test and Top-5 accuracy test were at their highest at 90.96% and 94.12%, respectively. Similarly, on three datasets with water ripple noise, the mean values of training accuracy reached a peak at 95.00%, while the mean values of the Top-1 accuracy test and Top-5 accuracy test were at their highest at 89.54% and 92.73%, respectively. These results demonstrate that the method showcases superior accuracy and enhanced robustness in handling original datasets and datasets with light reflection and water ripple noise.

## 1. Introduction

Fish are vital for humans as a protein source and for maintaining marine biodiversity [1]. However, they face challenges like overfishing, habitat destruction, and climate change.

Recognition of fish species benefits animal welfare, ecological protection, and wildlife support. Fish image recognition helps researchers understand fish behavior and improve habitats. It also aids in accurate population counting and the monitoring [2] of wild fish populations. Additionally, it enables rapid recognition of fish at customs and in markets, preventing the illegal trade of endangered species.

In blurry marine scenarios, fish species recognition is challenging, requiring accurate methods [3]. This contributes to surveys, population analyses, and the sustainable utilization of fish as a biological resource.

Underwater cameras are commonly used for fish surveys [4]. Unlike other methods, they minimize ecosystem impact and allow continuous recording of fish activity. However, limitations include a restricted field of view and factors like water turbidity, lighting conditions, and flow magnitude that affect image quality and recognition accuracy.

Previous research mainly focused on high-resolution fish recognition [5]. However, practical scenarios often feature blurry images due to water quality [6], relative movement [7] between the shooting device and the fish, water ripples [8], and light reflection [9]. This poses significant challenges for fish recognition in real-life situations.

In order to overcome the challenges mentioned above, a method of fish image recognition in blurry scenarios based on the diffusion model and attention [10,11,12] mechanism, DiffusionFR, is proposed herein. DiffusionFR offers a comprehensive set of technical solutions for fish recognition in blurry scenarios. It shows the selection and application of this correcting technique.

The main contribution list of this paper is summarized as follows:

(1) A two-stage diffusion model for fish recognition in blurry scenarios, TSD, was designed to maximize the removal of bad, blurry, and otherwise inadequate effects in fish images.
(2) A learnable attention module, LAM, was designed to ensure that the semantic features learned at the end of the network can distinguish fish for fine-grained recognition.
(3) A method for fish image recognition in blurry scenarios that synthesizes TSD and LAM, DiffusionFR, was proposed to present a complete solution for fish image recognition in blurry scenarios, and the selection and application of this correcting technique are presented herein.
(4) A dataset of fish images in blurry scenarios, BlurryFish, was constructed and used to validate the effectiveness of DiffusionFR, and integrated the bad, blurry, and otherwise inadequate images from the publicly available dataset Fish4Knowledge.

The structure of this paper is as follows. In Section 2, we review the relevant works on fish species recognition. Section 3 provides a detailed explanation of the key concepts and methodology used in this study. This includes the main ideas behind the method, DiffusionFR, the two-stage diffusion model (TSD), the learnable attention module (LAM), the modified ResNet as the recognition network, the dataset, and the experimental design. Moving on to Section 4, we present the treatment and analysis of the experimental findings. In Section 5, we thoroughly discuss the implications and significance of the results. Finally, in Section 6, we summarize the essential findings and draw conclusions based on the research conducted in this paper.

## 2. Background

Previous studies on fish species recognition commonly used different deep neural network architectures or employed layered and phased strategies.

Numerous studies on fish recognition have utilized various deep neural networks, such as CNN, Tripmix-Net, DAMNet, MobileNetv3, and VGG16. Villon et al. [13] employed CNN to enhance the accuracy of coral reef fish recognition by using rule-based techniques. They achieved a model accuracy of 94.9%, surpassing manual accuracy. Similarly, Villon et al. [14] used a convolutional neural network to analyze images from social media, providing support in monitoring rare megafauna species. Li et al. [15] proposed Tripmix-Net, a fish image classification model that incorporates multiscale network fusion. Qu et al. [16] introduced DAMNet, a deep neural network with a dual-attention mechanism for aquatic biological image classification. However, due to the incorporation of the dual-attention mechanism, the DAMNet model may exhibit a relatively higher level of complexity. Meanwhile, Alaba et al. [17] developed a model using the MobileNetv3-large and VGG16 backbone networks for fish detection. However, their method still encounters certain challenges, such as dealing with low-light conditions, noise, and the limitations posed by low-resolution images.

A hierarchical and phased approach to fish target recognition refers to dividing the recognition process into multiple phases and levels. Liang et al. [18] divided the recognition process into multiple stages to enhance accuracy and robustness. However, their method suffers from a high number of parameters and computational complexity, which can make the training process extremely time-consuming. Similarly, Ben et al. [5] proposed a hierarchical CNN classification method for automatic fish recognition in underwater environments.

In blurry scenarios [19], intelligent fish image recognition technology aims to improve image clarity using image processing techniques. These techniques include image denoising, image enhancement, and image alignment. Image denoising [20] reduces noise in the image using filters. Image enhancement [21] improves clarity through techniques like histogram equalization. Image alignment [22] addresses image blurring through registration. Neural heuristic video systems [23] analyze video frames automatically using heuristic algorithms, extending image analysis to video analysis. The bilinear pooling with poisoning detection (BPPD) module [24] utilizes bilinear pooling of convolutional neural networks. This algorithm combines data from two networks through bilinear pooling to achieve improved classification accuracy. Intelligent fish image recognition technology utilizes the diffusion model to deblur images. This model enhances image quality, recovers lost information, and improves feature extraction. As a result, it provides better inputs for subsequent image recognition tasks, significantly improving the accuracy of fish image recognition in blurry scenarios [25].

## 3. Materials and Methods

### 3.1. Main Ideas

**Figure 1:**
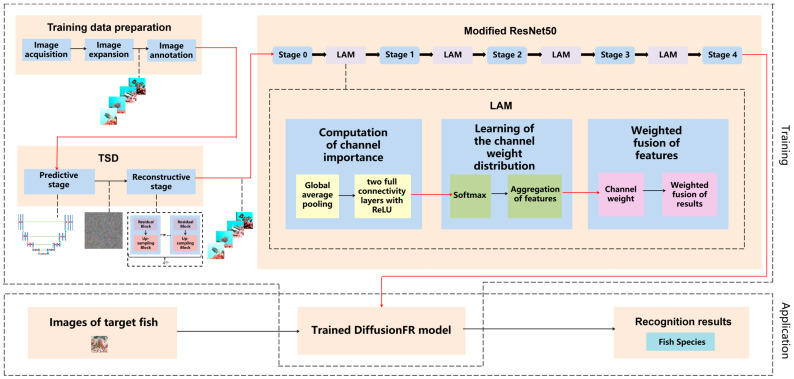
The structure for DiffusionFR, consisting of TSD, LAM, and the modified ResNet50.

Figure 1 presents the framework of the method based on the diffusion model and attention mechanism for fish image recognition in blurry scenarios, DiffusionFR. The framework visually illustrates the selection and application of the correction technique.

The main ideas behind DiffusionFR can be summarized as follows:

(1) Two-stage diffusion (TSD): This model consists of two stages—the predictive stage and the reconstructive stage. In the predictive stage, a U-Net structure generates feature probability maps for the bad, blurry, and otherwise inadequate fish images. Each pixel in the maps represents the probability of belonging to a specific class of fish image. In the reconstructive stage, four identical modules comprising a residual block and an up-sampling block are employed to convert the feature probability maps into clear fish images.
(2) Learnable attention module (LAM): The attention mechanism in DiffusionFR comprises three processes—the computation of channel importance, the learning of channel weight distribution, and the weighted fusion of features. The computation of channel importance involves global average pooling and two fully connected layers with ReLU activation. The learning of channel weight distribution includes SoftMax and the aggregation of features. Finally, the weighted fusion of features incorporates the channel weight and performs a weighted fusion of the results.
(3) Modifying ResNet as the recognition network: In DiffusionFR, the ResNet feature extraction network is modified by adding the LAM between each pair of adjacent stages. This modification aims to minimize the loss of accuracy, train a more precise recognition model, and enhance recognition accuracy for fish images in blurry scenarios.

### 3.2. Two-Stage Diffusion (TSD)

Recently, deep neural network-based diffusion models [26,27,28] have become popular for image denoising and super-resolution. These models utilize the capabilities of deep learning to learn image features and predict image evolution. As a result, they can quickly and efficiently denoise and enhance images.

**Figure 2:**
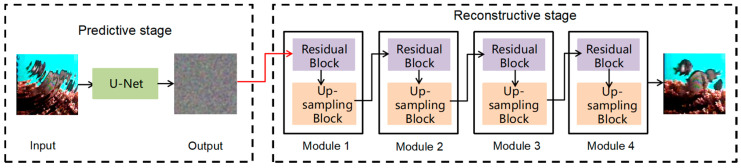
Structure of two-stage diffusion (TSD), including a predictive stage and a reconstructive stage.

The proposed TSD method in this study consists of two stages: a predictive stage and a reconstructive stage. The predictive stage detects fish image features in blurry images, while the reconstructive stage analyzes and processes diffusion data to address errors or deficiencies in the model. This stage significantly enhances the model’s accuracy and reliability. The entire TSD process is visually depicted in Figure 2.

The predictive stage of the proposed method takes the fish image as an input and generates a probability map, which represents the likelihood of each pixel belonging to a specific fish species category, as an output. This probability map provides valuable insights into the model’s classification probabilities for different fish species.

**Figure 3:**
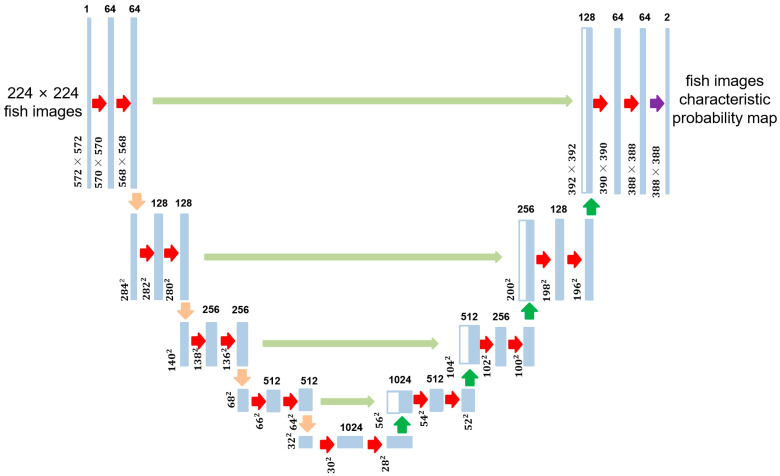
U-Net architecture, consisting of an encoder and a decoder.

To achieve this, the predictive stage utilizes the U-Net architecture [29], as shown in Figure 3. U-Net consists of symmetrical encoders and decoders. The encoder extracts image features using convolution and pooling operations, encoding the input image into a low-dimensional tensor. The decoder then reconstructs the encoder’s output into an image of the same dimensions as the input, with each pixel containing a probability value for the target category. To address information loss, U-Net incorporates jump connections that connect the feature maps of the encoder and decoder. It consists of four 2D convolutional layers and four maximum pooling layers, enabling the model to handle fish images of varying sizes and shapes within blurry images.

The restoration stage is responsible for generating the final restored image. It utilizes both the output image from the prediction stage and the input image.

**Figure 4:**
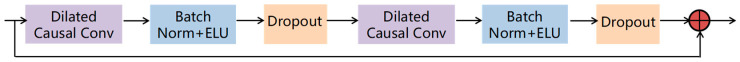
Residual Block, consisting of Dilated Causal Conv, BatchNorm, ELU, and Dropout.

The reconstructive stage is composed of four modules. Each module consists of a Residual Block [30] and Up-Sampling Block [31]. The Residual Block addresses issues of gradient vanishing and explosion during deep neural network training, as shown in Figure 4. It includes two convolutional layers and a jump connection, where the input is added directly to the output to form residuals. This helps the network capture the mapping relationship between inputs and outputs, improving the model’s performance and robustness. During model training, special attention is given to the error generated by the Up-Sampling Block in the network, as shown in Equation (1).

(1) $$q\left(x_{s}|x_{t},x_{0}\right)=N\left(x_{s}|\frac{1}{{g_{t0}}^{2}}\left(f_{s0}{g_{ts}}^{2}x_{0}+f_{ts}{g_{s0}}^{2}x_{t}\right),\frac{{g_{s0}}^{2}{g_{ts}}^{2}}{{g_{t0}}^{2}}I\right)$$

where $q\left(x_{s}|x_{t},x_{0}\right)$ denotes the conditional probability distribution of $x_{s};$ given conditions $x_{t}$ and $x_{0}$, the mean part of this is a series of linear combination terms including $f_{s0}$, $g_{ts}$, $f_{ts}$, and $g_{s0}$. In addition, $f_{ts}$ is a ratio indicating the relative scale that maps the input variable t to the input variable s, as shown in Equation (2), and $g_{ts}$ is computed from the scale parameters of the input variables t and s and is used to adjust the propagation process of the error, as shown in Equation (3).

(2) $$f_{ts}=\frac{f\left(t\right)}{f\left(s\right)}$$

(3) $$g_{ts}=\sqrt{{g\left(t\right)}^{2}-{f_{ts}}^{2}{g\left(s\right)}^{2}}$$

Equation (4) describes the gradual process of recovering the image from noise, as illustrated.

(4) $$x_{t}=\sqrt{{\bar{\alpha }}_{t}}x_{0}+{\bar{Z}}_{t-1}=\sqrt{{\bar{\alpha }}_{t}}x_{0}+\sqrt{1-{\bar{\alpha }}_{t}}Z,Z\sim N\left(0,I\right)$$

where $x_{t}$ denotes the image recovered at moment t, obtained by a linear combination of the initial image $x_{0}$ and the previous recovery result ${\bar{Z}}_{t-1}$. This linear combination uses a scaling factor $\sqrt{{\bar{\alpha }}_{t}}$ to adjust the contribution of the initial image and the previous recovery result. Meanwhile, the noise term Z is generated through a Gaussian distribution $N\left(0,I\right)$.

Equation (5) represents the inverse diffusion process from the recovered image $x_{t}$ back to the previously recovered result $x_{t-1}$.

(5) $$q\left(x_{t-1}|x_{t}{,x}_{0}\right)=N\left(x_{t-1};\frac{1}{\sqrt{\alpha_{t}}}x_{t}-\frac{\beta_{t}}{\sqrt{\alpha_{t\left(1-{\bar{\alpha }}_{t}\right)}}}Z,\frac{1-{\bar{\alpha }}_{t-1}}{1-{\bar{\alpha }}_{t}}\beta_{t}\right),Z\sim N\left(0,I\right)$$

where the conditional probability distribution $q\left(x_{t-1}|x_{t},x_{0}\right)$ represents the conditional probability distribution of $x_{t-1};$ given conditions $x_{t}$ and $x_{0}$, this conditional probability distribution is represented by a Gaussian distribution where the mean part contains a linear combination of $x_{t}$ and the noise term Z.

TSD implements batch normalization techniques and dropout layers to enhance the stability, convergence, and generalization of the model.

### 3.3. Learnable Attention Module (LAM)

**Figure 5:**
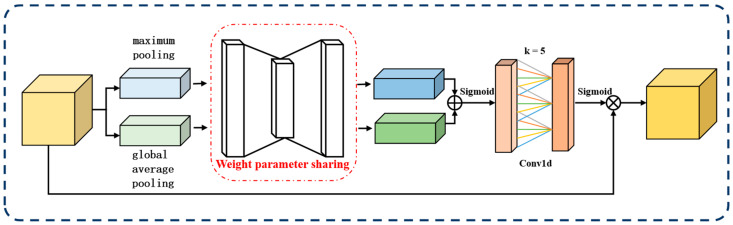
The framework structure of the learnable attention module (LAM).

In this paper, we propose LAM, which is based on the channel attention mechanism [32] (CAM) and depicted in Figure 5. Unlike CAM, LAM assigns weights to channels by learning the importance of features.

In DiffusionFR, the LAM consists of three steps. These steps include the computation of channel importance, learning of channel weight distribution, and weighted fusion of features. These steps are illustrated in Figure 1.

The first step is the computation [33] of channel importance. First, the global pooling values for each channel in the feature map F are extracted by a global average pooling or maximum pooling operation to obtain a C-dimensional vector Z. Then, Z is processed using a network architecture containing two fully connected layers and a ReLU activation function to generate a C-dimensional weight assignment vector k, which stores the weight assignments for each channel, as shown in Equation (6).

(6) $$k=\varphi \left(C\right)={\left|\frac{\log_{2}\left(C\right)}{\gamma }+\frac{b}{\gamma }\right|}_{odd}$$

The second step involves the learning [34] of the channel weight distribution. This distribution determines the significance of each feature map channel. To compute the channel weights, we use the softmax function to map the values in the weight vector between 0 and 1. This ensures that the sum of all weights equals 1, representing the weight of each channel. To gather global information about the channels, we apply a global average pooling operation to the features, which is represented by Equation (7).

(7) $$y=\frac{1}{H\times W}\sum_{a}^{H}\sum_{b}^{W}x_{i}(a,b)$$

In the formula, $x_{i}$ represents the ith feature map of input size H×W, and y represents the global feature. In the softmax function, each feature vector element is mapped to a value between 0 and 1. With this mapping, the model can determine how much each channel contributes relative to the overall feature map.

The third step involves the weighted fusion [35] of features. Each channel in the feature map is weighted and fused based on their assigned weights. Firstly, weights are assigned to each channel and applied to their corresponding features. Then, the features of all channels are proportionally weighted and fused to generate a feature map adjusted by the attention mechanism. By incorporating the LAM, the network can dynamically adjust the contribution of each channel, improving its robustness and generalization ability. This attention mechanism enables the network to disregard irrelevant information (weights close to 0) and prioritize important features essential for successful task completion.

### 3.4. Modifying ResNet as the Recognition Network

DiffusionFR selected ResNet50 as the base network after comparing ResNet34 [36], ResNet50 [37], and ResNet101 [38].

ResNet50 has a layered architecture that enables it to learn hierarchical representations of input data. Lower layers capture low-level features, while higher layers capture intricate patterns and relationships. The pooling layer reduces spatial dimensionality, improving computational efficiency and translation invariance while mitigating overfitting. By leveraging ResNet50’s transfer learning, this provides a solid foundation for the probabilistic graph generation task.

**Figure 6:**
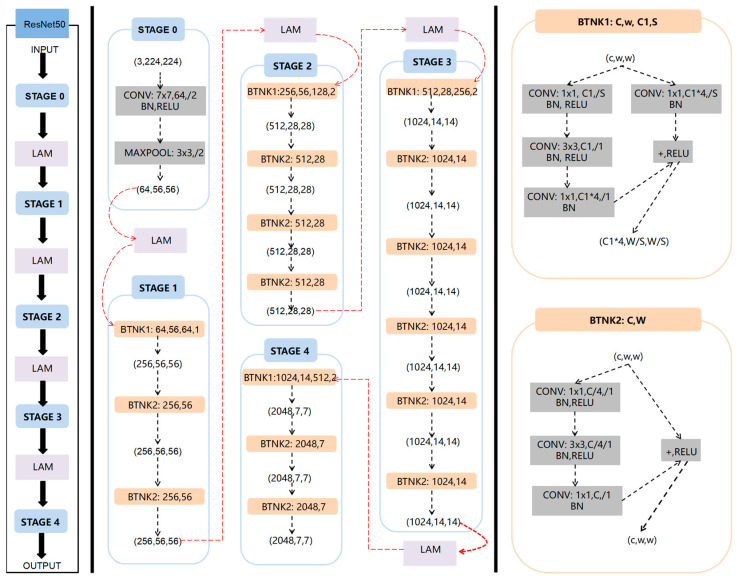
Structure of the modified ResNet50 with the LAM added between every two neighboring stages.

However, in images with complex backgrounds or noise, ResNet50 may unintentionally focus on less relevant regions, impacting model performance. To address this, an attention mechanism is introduced to dynamically adjust feature map weights based on different parts of the input data. This helps prioritize crucial features, enhancing accuracy and generalization capabilities. Therefore, the DiffusionFR approach modifies ResNet50 by incorporating LAM into the network. LAM is added between each pair of adjacent stages, as shown in Figure 6.

### 3.5. Dataset

This paper introduces BlurryFish, a fish image dataset created by integrating blurred images from the publicly available dataset Fish4Knowledge. The construction process involved the following steps:

(1) Data Collection

**Figure 7:**
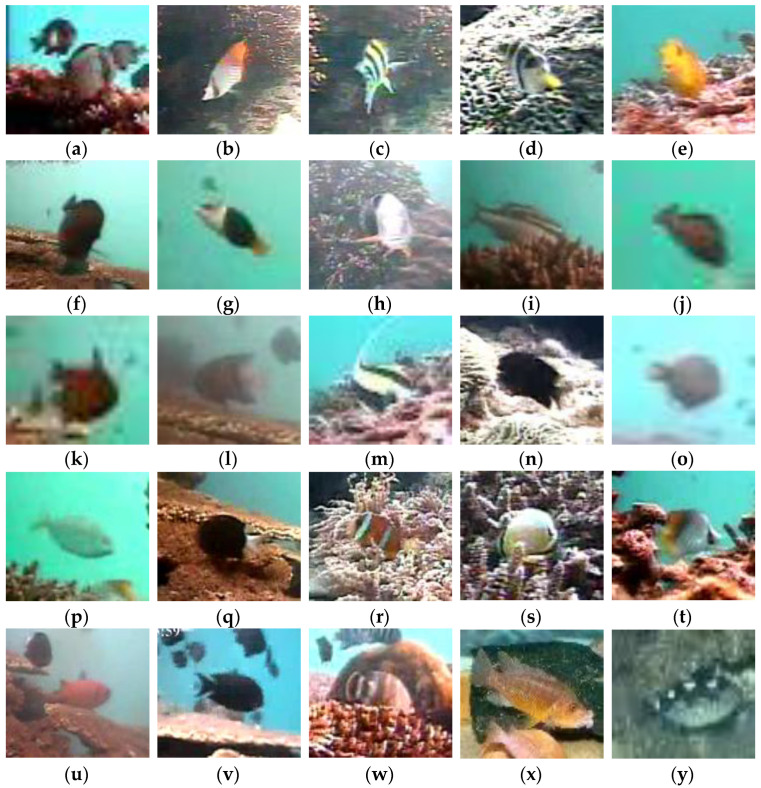
Fish species in the dataset. (**a**) Dascyllus reticulatus; (**b**) Neoniphon sammara; (**c**) Abudefduf vaigiensis; (**d**) Canthigaster valentini; (**e**) Pomacentrus moluccensis; (**f**) Zebrasoma scopas; (**g**) Hemigymnus melapterus; (**h**) Lutjanus fulvus; (**i**) Scolopsis bilineata; (**j**) Scaridae; (**k**) Pempheris vanicolensis; (**l**) Plectroglyphidodon dickii; (**m**) Zanclus cornutus; (**n**) Neoglyphidodon nigroris; (**o**) Balistapus undulatus; (**p**) Siganus fuscescens; (**q**) Chromis chrysura; (**r**) Amphiprion clarkii; (**s**) Chaetodon lunulatus; (**t**) Chaetodon trifascialis; (**u**) Myripristis kuntee; (**v**) Acanthurus nigrofuscus; (**w**) Hemigymnus fasciatus; (**x**) Abactochromis labrosus; and (**y**) Abalistes stellaris.

The datasets used in this paper are from three sources. The first source is the publicly available dataset Fish4Knowledge, consisting of realistically shot images. The second source is a field-photographed dataset that prioritizes challenging scenarios like low-light conditions and inclement weather to ensure representative fish images. The third source is fish images from Internet, which we organized and classified. The dataset comprises 25 fish species, and Figure 7 displays these species and example images.

(2) Data Cleaning

The collected fish images underwent a cleaning process to ensure their quality and reliability. This involved eliminating invalid samples and duplicate samples.

(3) Dataset Partition

The dataset was divided into three sets for the experiments: the training set, the validation set, and the test set. This division follows the leave-out method [39] and maintains an 8:1:1 ratio. The goal was to ensure that all sets included pictures of the same fish species, as well as similar scenarios and angles.

(4) Data Enhancement

The collected dataset has an interclass balance problem [40] due to the varying number of pictures for each fish species. This can result in lower recognition accuracy for less common fish species if the dataset is directly used for training. To address this issue, standard data enhancement methods were employed, including panning, cropping, rotating, mirroring, flipping, and brightness adjustment. These operations generated additional image samples, enhancing the model’s robustness, generalization ability, and recognition accuracy for smaller fish species. Table 1 shows that the initial BlurryFish dataset contained 2754 bad, blurry, and otherwise inadequate fish images. However, after applying data enhancement techniques, the dataset expanded to 35,802 bad, blurry, and otherwise inadequate fish images, as shown in Table 2.

(5) Data Annotation

To create valuable training and testing sets from the image dataset, each image in the fish image dataset was labeled with associated fish species data. We utilized the graphical interface labeling software, LabelImg (v 1.8.5), to annotate the fish images and generate XML files. Although DiffusionFR does not impose any restrictions on the resolution and other parameters of the dataset images, we uniformly converted the dataset to RGB images with a resolution of 224 × 224. These images were then stored in the PASCAL VOC data format.

### 3.6. Experimental Design

#### 3.6.1. Experimental Environment Configuration

PyTorch, a deep learning framework, was employed to evaluate DiffusionFR. The specific experimental software and hardware configurations are detailed in Table 3.

#### 3.6.2. Evaluation Indicators

To evaluate the model’s performance in classifying fish images in blurry scenarios, accuracy and Top-k accuracy were used as evaluation metrics. The experimental data was processed using Python code and analyzed using Excel software (12.1.0.16250).

(1) Accuracy

Accuracy is a metric that measures the proportion of correctly predicted samples compared to the total number of instances. It is calculated using Equation (8).

(8) $$Accuracy=\frac{TN+TP}{TN+FP+TP+FN}\times 100\%$$

where TN denotes true negative, TP denotes true positive, FP denotes false positive, and FN denotes false negative.

(2) Top-k Accuracy

The top-k accuracy rate measures the proportion of samples where at least one of the top-k predictions matches the true label, compared to the total number of samples. In this study, we use Top-1 accuracy and Top-5 accuracy as model criteria. Equation (9) demonstrates the calculation of the top-k accuracy.

(9) $$\begin{array}{cc} Top-k\;Accuracy & \\ & =number\;of\;samples\;correctly\;predicted\\ & /the\;total\;number\;of\;samples\times 100\% \end{array}$$

Here, k can be any positive integer, and it is common to have top−1 and top−5 accuracy rates, which indicate the accuracy in the highest confidence prediction and the first five highest confidence predictions, respectively.

#### 3.6.3. Parameters of Experiments

In this section, we conduct comparative experiments for each module of DiffusionFR. During training, the model parameters of DiffusionFR were continuously adjusted to minimize prediction errors. This was achieved using optimization algorithms and loss functions. After several iterations, the hyperparameters of the DiffusionFR model were determined based on commonly used empirical values. The finalized hyperparameters can be found in Table 4.

#### 3.6.4. Schemes of Experiments

(1) Comparison of Backbone Networks

DiffusionFR’s backbone network was assessed using the original dataset. The analysis included various backbone networks such as ResNet50, VGG16, MobileNetv3, Tripmix-Net, ResNeXt, DAMNet, ResNet34, ResNet101, EfficientNet [41], neuro-heuristic, bilinear pooling with poisoning detection (BPPD), and CNN(r1, r2).

(2) Comparison of Attention Mechanisms

Comparative experiments were conducted to assess the impact of attention mechanisms on the algorithm. The evaluated attention mechanisms included LAM, CBAM [42], CCA [43], and SE [44].

(3) Comparison of Diffusion Models

To assess the impact of the diffusion model proposed in this paper on the final recognition performance, we conducted a comparative experiment. This experiment involved two deblurring methods: the diffusion module proposed in this paper and the Gaussian denoising module.

(4) Effect of Light Reflection Noise on Recognition Performance

The datasets were labeled according to the light reflection noise added. For instance, D_0_E_0_ signifies the original dataset without any added noise, while D_0.6_E_100_ represents the dataset with light reflection noise, where the light diameter is 0.6 cm and the light intensity is 100 Lux, added to D_0_E_0_. This naming convention is used for other datasets as well.

**Figure 8:**
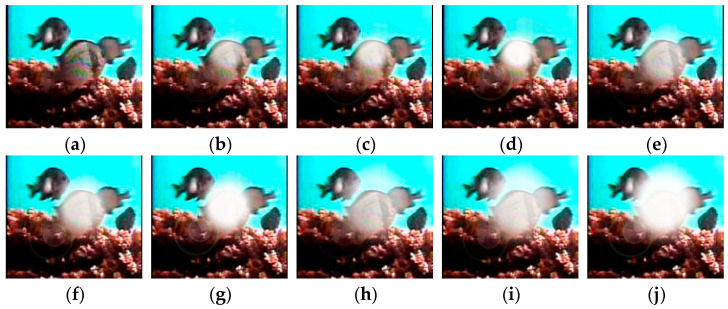
Example of fish pictures with added light reflection noise. (**a**) D_0_E_0_; (**b**) D_0.6_E_100_; (**c**) D_0.6_E_250_; (**d**) D_0.6_E_400_; (**e**) D_0.8_E_100_; (**f**) D_0.8_E_250_; (**g**) D_0.8_E_400_; (**h**) D_1.0_E_100_; (**i**) D_1.0_E_250_; and (**j**) D_1.0_E_400_.

Nine datasets were created by categorizing the light reflection noise based on different light diameters and intensities. These datasets are named as D_0.6_E_100_, D_0.6_E_250_, D_0.6_E_400_, D_0.8_E_100_, D_0.8_E_250_, D_0.8_E_400_, D_1.0_E_100_, D_1.0_E_250_, and D_1.0_E_400_. Table 5 provides an overview of the data volume for the fish image dataset with added light reflection noise. An example of this dataset is shown in Figure 8.

We conducted a comparative analysis on datasets with light reflection noise to assess the effectiveness of using corrected fish images for species-specific fish recognition.

(5) Effect of Water Ripple Noise on Recognition Performance

To add water ripple noise to the dataset and generate the water ripple effect, the following steps and Equations were used. First, an empty array X of the same size as the original image was created to store the generated water ripple effect. Next, offsets (including offset_x and offset_y) were calculated for each pixel based on the amplitude (A) and frequency (F) by iterating through each pixel in a loop. Then, the pixel values corresponding to these offsets were assigned to each pixel of the empty array X, generating the water ripple effect. Finally, the resulting water ripple effect was overlaid onto the original image, creating the final image with water ripples. Equations involved are shown in (10)–(14).

The offset was calculated using Equations (10) and (11).

(10) $$\text{offset\_x}=A*\sin\left(2*\pi *y_{i}*F\right)$$

(11) $$\text{offset\_y}=A*\cos\left(2*\pi *x_{i}*F\right)$$

where $\left(x_{i},y_{i}\right)$ denotes the coordinates of a pixel point in the image, F is the frequency of the water ripple, and A is the amplitude of the water ripple.

The pixel assignment of array X is calculated using Equations (12) and (13).

(12) $$X\left[x_{i}\right]=\left(x_{i}+\text{offset\_x}\right)\%width$$

(13) $$X\left[y_{i}\right]=\left(y_{i}+\text{offset\_y}\right)\%height$$

where width and height are the width and height of the image, respectively.

The final image generation is calculated using Equation (14).

(14) img_with_ripples=img+X

where img denotes the original image, and img_with_ripples denotes the final image with water ripples.

The datasets were labeled according to the water ripple noise added. For instance, F_0_A_0_ signifies the original dataset without any added noise, while F_0.04_A_2_ indicates the dataset with water ripple noise having a frequency of 0.04 and an amplitude of 2 added to F_0_A_0_. This naming convention is used for other datasets as well.

**Figure 9:**
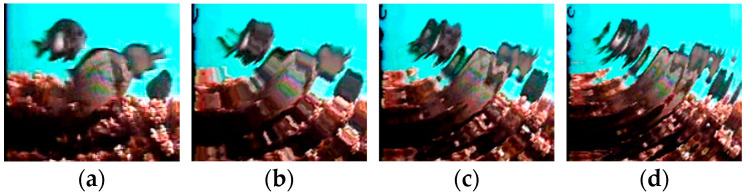
Example of fish pictures with added water ripple noise. (**a**) F_0_A_0_; (**b**) F_0.04_A_2_; (**c**) F_0.06_A_6_; and (**d**) F_0.08_A_10_.

Water ripple noise can be classified based on the frequency and amplitude of the water ripples. Increasing the frequency and amplitude results in a higher offset and greater oscillation in the generated water waves. In this study, the water ripple noise was categorized into three groups: F_0.04_A_2_, F_0.06_A_6_, and F_0.08_A_10_, primarily based on their frequency and amplitude. Table 6 provides an overview of the data volume of the fish image dataset with the addition of water ripple noise, while Figure 9 offers an illustrative example.

We conducted a comparative analysis of datasets with water ripple noise to assess the effectiveness of using corrected fish images for species-specific fish recognition.

We conducted Experiments 1 through 5 to assess the impact of different backbone networks, attention mechanisms, diffusion models, as well as light reflection noise and water ripple noise on recognition performance. These experiments were evaluated using three metrics: training accuracy, the Top-1 accuracy test, and the Top-5 accuracy test. The objective was to comprehensively evaluate their recognition performance and analyze the results.

## 4. Results

In this study, the BlurryFish dataset was used to perform comparative experiments on the key innovations of the proposed methodology.

### 4.1. Comparison of Backbone Networks

This study compared and analyzed the backbone network of DiffusionFR. For example, DiffusionFR_VGG16 refers to using VGG16 instead of ResNet50 as the backbone network in DiffusionFR. Similar comparisons were made with other backbone networks. The results of these comparisons can be found in Table 7.

Table 7 displays the performance metrics of DiffusionFR on the original dataset. The training accuracy is 97.55%. The corresponding Top-1 accuracy test score was 92.02%, and the Top-5 accuracy test score was 95.17%. These values indicate that DiffusionFR outperforms other methods in terms of accuracy. These values also demonstrate that DiffusionFR, with ResNet50 as the chosen backbone network, has a higher potential for achieving superior recognition performance.

### 4.2. Comparison of Attention Mechanisms

To evaluate the impact of the attention mechanism on the algorithm, a comparative experiment was conducted, as shown in Table 8. The experiment compared the performance of DiffusionFR with DiffusionFR without any attention mechanism, referred to as DiffusionFR_noA. Furthermore, classical attention methods were used as substitutes for LAM. For example, DiffusionFR_CBAM incorporated CBAM as the attentional method in DiffusionFR. The results of these comparisons are presented in Table 8.

Table 8 shows that the training accuracy of DiffusionFR on the original dataset was 97.55%. The corresponding Top-1 accuracy test score was 92.02%, and the Top-5 accuracy test score was 95.17%. It is important to note that all these metrics outperform the performance of other methods. This establishes DiffusionFR as the method with the most effective recognition capability.

### 4.3. Comparison of Diffusion Models

The final recognition results for the diffusion model proposed in this paper were obtained through experiments, as presented in Table 9. This table includes the performance of DiffusionFR, DiffusionFR_noTSD, and DiffusionFR_Gaussian. DiffusionFR_noTSD refers to the method where the TSD was removed from the proposed method, and DiffusionFR_Gaussian involves using Gaussian denoising [45] instead of the TSD. The results of these methods are compared in Table 9.

Table 9 presents the performance metrics of DiffusionFR on the original dataset. The training accuracy of DiffusionFR is recorded as 97.55%. The corresponding Top-1 accuracy test and Top-5 accuracy test scores are reported as 92.02% and 95.17%, respectively. It is important to note that all these metrics outperform the performance of other methods. This establishes DiffusionFR as the method with the most effective recognition capability.

### 4.4. Effect of Light Reflection Noise on Recognition

**Figure 10:**
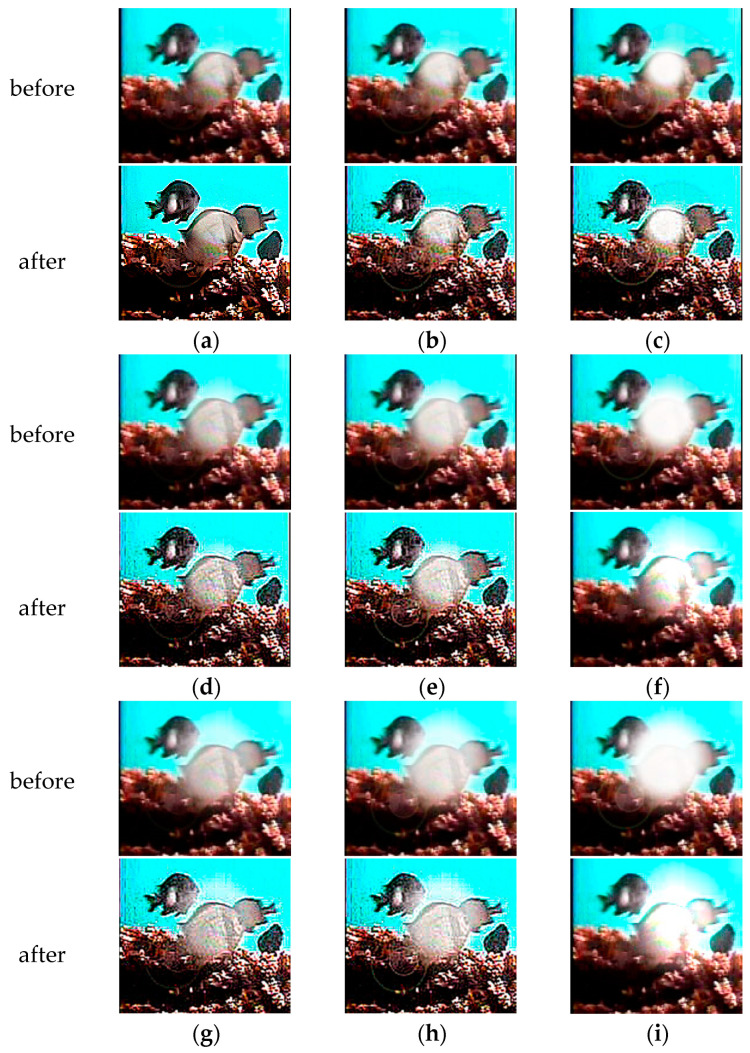
Comparison of images before and after TSD deblurring of light reflection noise. (**a**) D_0.6_E_100_; (**b**) D_0.6_E_250_; (**c**) D_0.6_E_400_; (**d**) D_0.8_E_100_; (**e**) D_0.8_E_250_; (**f**) D_0.8_E_400_; (**g**) D_1.0_E_100_; (**h**) D_1.0_E_250_; and (**i**) D_1.0_E_400_.

We performed a comparative analysis using DiffusionFR’s backbone network on datasets with light reflection noise to evaluate the usability of corrected fish images for species-specific fish recognition. The results of this analysis are presented in Table 10. TSD’s effectiveness in processing fish images with light reflection noise is visually demonstrated in Figure 10. The presence of TSD reduces the noise before deblurring, thereby preserving critical features for accurate recognition. Additionally, TSD performs better in handling light reflection noise compared to water ripple noise.

In Table 10, the mean value of the training accuracy of DiffusionFR on the nine datasets (D_0.6_E_100_, D_0.6_E_250_, D_0.6_E_400_, D_0.8_E_100_, D_0.8_E_250_, D_0.8_E_400_, D_1.0_E_100_, D_1.0_E_250_, and D_1.0_E_400_) with added light reflection noise was 86.85%. The mean value of the Top-1 accuracy test was 81.87%, and the mean value of the Top-5 accuracy test was 84.71%. These values indicate that DiffusionFR outperforms other methods in terms of accuracy. These values also demonstrate that DiffusionFR, with ResNet50 as the chosen backbone network, has a higher potential for achieving superior recognition performance.

### 4.5. Effect of Water Ripple Noise on Recognition

**Figure 11:**
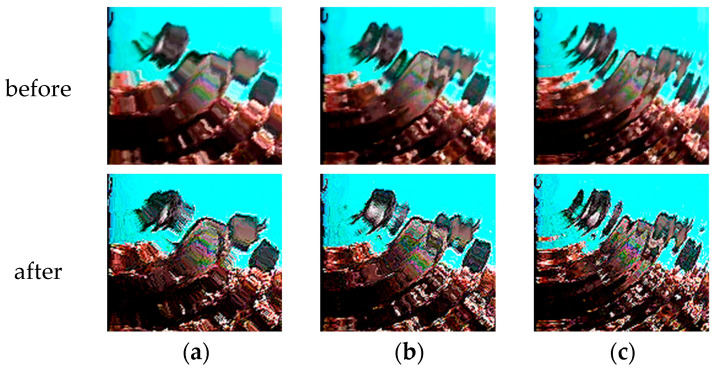
Comparison of images before and after TSD deblurring of water ripple noise. (**a**) F_0.04_A_2_; (**b**) F_0.06_A_6_; (**c**) F_0.08_A_10_.

We conducted a comparative analysis using DiffusionFR’s backbone network on datasets with water ripple noise to evaluate the usability of corrected fish images for species-specific fish recognition. The results of this analysis can be found in Table 11. Figure 11 provides a visual representation of TSD’s ability to process fish images containing water ripple noise. TSD effectively reduces the frequency and intensity of water ripple noise in the images before deblurring, mitigating its impact on the critical feature extraction capability of the DiffusionFR model. This ensures that the fish image before deblurring can accurately show ID characters.

In Table 11, the mean value of the training accuracy of DiffusionFR on the three datasets (F_0.04_A_2_, F_0.06_A_6_, and F_0.08_A_10_) with added water ripple noise is 95.00%. The mean value of the Top-1 accuracy test was 89.54%, and the mean value of the Top-5 accuracy test was 92.73%. These values indicate that DiffusionFR outperforms other methods in terms of accuracy. These values also demonstrate that DiffusionFR, with ResNet50 as the chosen backbone network, has a higher potential for achieving superior recognition performance.

## 5. Discussion

Based on the analysis of Table 7, Table 8, Table 9, Table 10 and Table 11, we have drawn several significant conclusions. Firstly, ResNet50 performs better than other backbone networks when selected as the backbone network for DiffusionFR. Compared to ResNet34 and ResNet101, the deeper network structure of ResNet50 enables a more effective capture of intricate image features and mitigates the risk of gradient vanishing or explosion [46]. Additionally, ResNet50’s effective integration of the attention mechanism and the residual network approach contribute to its superior performance in propagating the model gradient.

Furthermore, a comparison between DiffusionFR and DiffusionFR_noA reveals that DiffusionFR outperforms DiffusionFR_noA in terms of training accuracy and accuracy on the test set. This indicates that DiffusionFR is capable of capturing crucial features and achieving more accurate classification and prediction. DiffusionFR also demonstrates superior performance compared to other standard attention methods, further validating the effectiveness of the incorporated LAM.

Moreover, DiffusionFR exhibits remarkable results among the compared methods, achieving superior performance in terms of training accuracy and accuracy on the test set. The proposed TSD approach for fish recognition in blurry scenarios proves to be highly effective. DiffusionFR’s end-to-end integrated framework [47] for denoising and recognition surpasses a two-stage scheme by leveraging the interrelationships between these tasks. It enhances accuracy and stability by efficiently handling noise [48] and blur [49] information.

Additionally, the impact of light reflection noise and water ripple noise on recognition performance is evident from the analysis. Increasing light amplitude, light diameter, frequency, and amplitude of water ripples in the datasets leads to a decreasing trend in the training accuracy, Top-1 test accuracy, and Top-5 test accuracy of the same backbone network method. This highlights the significant role of light reflection and water ripples in recognition performance and reinforces the usability of corrected fish images for species-specific recognition even in the presence of these noise scenarios.

In comparing the neuro-heuristic analysis of video and bilinear pooling with poisoning detection (BPPD) to the DiffusionFR method, it becomes clear that DiffusionFR outperforms these approaches. While recent advancements in the neural network field have shown progress, DiffusionFR exhibits superior performance, even when compared to CNN(r1, r2).

## 6. Conclusions

In this study, we propose a method called DiffusionFR, which combines the diffusion model and attention mechanism to address the challenge of fish image recognition in blurry scenarios. The approach involves deblurring fish scene pictures using a two-stage diffusion network model, TSD, to restore clarity. Furthermore, a learnable attention module, LAM, was incorporated to enhance the accuracy of fish recognition.

DiffusionFR achieves the highest mean values of training accuracy, Top-1 test accuracy, and Top-5 test accuracy, at 94.91% on the original dataset. It also maintains the highest mean values of accuracy at 94.65% on the datasets with added light reflection noise and 92.84% on the datasets with added water ripple noise.

The effectiveness of DiffusionFR is evident from its superior performance compared to other approaches that use different backbone networks, attention mechanisms, and Gaussian denoising. DiffusionFR proves to be more accurate and robust, making it applicable in various underwater applications such as underwater photography, underwater detection, and underwater robotics.

Although this study successfully improves fish image recognition in blurry scenarios, there is still room for improvement due to the complex and uncertain nature of the marine environment. Additionally, the recognition of overlapping and occluded regions in natural fish scenarios needs further exploration. It is essential to construct relevant datasets, refine the network model, and conduct comprehensive studies to contribute effectively to fish conservation and related industries in the future.

## Figures and Tables

**Table 1:** Number of fish images in the BlurryFish dataset before data enhancement.

ID	Name of Fish	Training Set	Validation Set	Test Set	Total
1	*Dascyllus reticulatus*	91	12	12	115
2	*Neoniphon sammara*	84	11	11	106
3	*Abudefduf vaigiensis*	85	10	10	105
4	*Canthigaster valentini*	88	11	11	110
5	*Pomacentrus moluccensis*	94	12	12	118
6	*Zebrasoma scopas*	85	11	11	107
7	*Hemigymnus melapterus*	84	10	10	104
8	*Lutjanus fulvus*	83	10	10	103
9	*Scolopsis bilineata*	86	11	11	108
10	*Scaridae*	92	11	11	114
11	*Pempheris vanicolensis*	82	10	10	102
12	*Plectroglyphidodon dickii*	85	11	11	107
13	*Zanclus cornutus*	95	12	12	119
14	*Neoglyphidodon nigroris*	85	10	10	105
15	*Balistapus undulatus*	89	11	11	111
16	*Siganus fuscescens*	92	11	11	114
17	*Chromis chrysura*	92	12	12	116
18	*Amphiprion clarkii*	84	11	11	106
19	*Chaetodon lunulatus*	91	12	12	115
20	*Chaetodon trifascialis*	95	12	12	119
21	*Myripristis kuntee*	90	11	11	112
22	*Acanthurus nigrofuscus*	87	11	11	109
23	*Hemigymnus fasciatus*	82	10	10	102
24	*Abactochromis labrosus*	89	11	11	111
25	*Abalistes stellaris*	92	12	12	116
Total		2202	276	276	2754

**Table 2:** Number of fish images in the BlurryFish dataset after data enhancement.

ID	Name of Fish	Training Set	Validation Set	Test Set	Total
1	*Dascyllus reticulatus*	1195	150	150	1495
2	*Neoniphon sammara*	1102	138	138	1378
3	*Abudefduf vaigiensis*	1093	136	136	1365
4	*Canthigaster valentini*	1144	143	143	1430
5	*Pomacentrus moluccensis*	1228	153	153	1534
6	*Zebrasoma scopas*	1113	139	139	1391
7	*Hemigymnus melapterus*	1082	135	135	1352
8	*Lutjanus fulvus*	1071	134	134	1339
9	*Scolopsis bilineata*	1124	140	140	1404
10	*Scaridae*	1186	148	148	1482
11	*Pempheris vanicolensis*	1060	133	133	1326
12	*Plectroglyphidodon dickii*	1113	139	139	1391
13	*Zanclus cornutus*	1237	155	155	1547
14	*Neoglyphidodon nigroris*	1093	136	136	1365
15	*Balistapus undulatus*	1155	144	144	1443
16	*Siganus fuscescens*	1186	148	148	1482
17	*Chromis chrysura*	1206	151	151	1508
18	*Amphiprion clarkia*	1102	138	138	1378
19	*Chaetodon lunulatus*	1195	150	150	1495
20	*Chaetodon trifascialis*	1237	155	155	1547
21	*Myripristis kuntee*	1164	146	146	1456
22	*Acanthurus nigrofuscus*	1133	142	142	1417
23	*Hemigymnus fasciatus*	1060	133	133	1326
24	*Abactochromis labrosus*	1155	144	144	1443
25	*Abalistes stellaris*	1206	151	151	1508
Total		28,640	3581	3581	35,802

**Table 3:** Experimental software and hardware configurations.

Item	Detail
GPU	NVIDIA GeForce RTX 3060
CPU	12th Gen Intel(R) Core(TM) i5-12400 2.50 GHz
RAM	16.0 GB
Operating system	Windows 11 64-bit
CUDA	CUDA 11.6
Python	Python 3.7.15

**Table 4:** Optimal hyperparameters.

Input Shape	Lr	Activation Function	Batch Size	Classifier	Optimizer	Epoch
224 × 224	0.002	ReLU	32	Softmax	Adam	100

**Table 5:** Fish Dataset with Added Light Reflection Noise.

ID	Name of Fish	D_0.6_E_100_	D_0.6_E_250_	D_0.6_E_400_	D_0.8_E_100_	D_0.8_E_250_	D_0.8_E_400_	D_1.0_E_100_	D_1.0_E_250_	D_1.0_E_400_	Total
1	*Dascyllus reticulatus*	115	115	115	115	115	115	115	115	115	1035
2	*Neoniphon sammara*	106	106	106	106	106	106	106	106	106	954
3	*Abudefduf vaigiensis*	105	105	105	105	105	105	105	105	105	945
4	*Canthigaster valentini*	110	110	110	110	110	110	110	110	110	990
5	*Pomacentrus moluccensis*	118	118	118	118	118	118	118	118	118	1062
6	*Zebrasoma scopas*	107	107	107	107	107	107	107	107	107	963
7	*Hemigymnus melapterus*	104	104	104	104	104	104	104	104	104	936
8	*Lutjanus fulvus*	103	103	103	103	103	103	103	103	103	927
9	*Scolopsis bilineata*	108	108	108	108	108	108	108	108	108	972
10	*Scaridae*	114	114	114	114	114	114	114	114	114	1026
11	*Pempheris vanicolensis*	102	102	102	102	102	102	102	102	102	918
12	*Plectroglyphidodon dickii*	107	107	107	107	107	107	107	107	107	963
13	*Zanclus cornutus*	119	119	119	119	119	119	119	119	119	1071
14	*Neoglyphidodon nigroris*	105	105	105	105	105	105	105	105	105	945
15	*Balistapus undulatus*	111	111	111	111	111	111	111	111	111	999
16	*Siganus fuscescens*	114	114	114	114	114	114	114	114	114	1026
17	*Chromis chrysura*	116	116	116	116	116	116	116	116	116	1044
18	*Amphiprion clarkii*	106	106	106	106	106	106	106	106	106	954
19	*Chaetodon lunulatus*	115	115	115	115	115	115	115	115	115	1035
20	*Chaetodon trifascialis*	119	119	119	119	119	119	119	119	119	1071
21	*Myripristis kuntee*	112	112	112	112	112	112	112	112	112	1008
22	*Acanthurus nigrofuscus*	109	109	109	109	109	109	109	109	109	981
23	*Hemigymnus fasciatus*	102	102	102	102	102	102	102	102	102	918
24	*Abactochromis labrosus*	111	111	111	111	111	111	111	111	111	999
25	*Abalistes stellaris*	116	116	116	116	116	116	116	116	116	1044
Total		2754	2754	2754	2754	2754	2754	2754	2754	2754	24,786

**Table 6:** Fish Dataset with Added Water Ripple Noise.

ID	Name of Fish	F_0.04_A_2_	F_0.06_A_6_	F_0.08_A_10_	Total
1	*Dascyllus reticulatus*	115	115	115	345
2	*Neoniphon sammara*	106	106	106	318
3	*Abudefduf vaigiensis*	105	105	105	315
4	*Canthigaster valentini*	110	110	110	330
5	*Pomacentrus moluccensis*	118	118	118	354
6	*Zebrasoma scopas*	107	107	107	321
7	*Hemigymnus melapterus*	104	104	104	312
8	*Lutjanus fulvus*	103	103	103	309
9	*Scolopsis bilineata*	108	108	108	324
10	*Scaridae*	114	114	114	342
11	*Pempheris vanicolensis*	102	102	102	306
12	*Plectroglyphidodon dickii*	107	107	107	321
13	*Zanclus cornutus*	119	119	119	357
14	*Neoglyphidodon nigroris*	105	105	105	315
15	*Balistapus undulatus*	111	111	111	333
16	*Siganus fuscescens*	114	114	114	342
17	*Chromis chrysura*	116	116	116	348
18	*Amphiprion clarkii*	106	106	106	318
19	*Chaetodon lunulatus*	115	115	115	345
20	*Chaetodon trifascialis*	119	119	119	357
21	*Myripristis kuntee*	112	112	112	336
22	*Acanthurus nigrofuscus*	109	109	109	327
23	*Hemigymnus fasciatus*	102	102	102	306
24	*Abactochromis labrosus*	111	111	111	333
25	*Abalistes stellaris*	116	116	116	348
Total		2754	2754	2754	8262

**Table 7:** Accuracies of Different Feature Extraction Networks.

Model	Training (%)	Top-1 Test (%)	Top-5 Test (%)
DiffusionFR	97.55	92.02	95.17
DiffusionFR_VGG16	91.78	86.38	89.48
DiffusionFR_MobileNetv3	93.05	87.55	90.60
DiffusionFR_Tripmix-Net	93.43	88.07	91.21
DiffusionFR_ResNeXt	94.32	88.90	91.98
DiffusionFR_DAMNet	93.80	88.26	91.38
DiffusionFR_ResNet34	96.22	90.80	93.85
DiffusionFR_ResNet101	94.35	88.92	91.72
DiffusionFR_EfficientNet	96.10	90.48	93.38
DiffusionFR_neuro-heuristic	96.82	91.25	94.27
DiffusionFR_BPPD	97.02	91.49	94.63
DiffusionFR_CNN(r1, r2)	97.33	91.69	94.80

**Table 8:** Accuracies of Different Attention Mechanisms for LAMs.

Model	Training (%)	Top-1 Test (%)	Top-5 Test (%)
DiffusionFR	97.55	92.02	95.17
DiffusionFR_noA	95.31	89.98	93.06
DiffusionFR_CBAM	96.50	91.10	94.22
DiffusionFR_CCA	97.03	91.52	94.57
DiffusionFR_SE	96.05	90.59	93.70

**Table 9:** Accuracies of Different Diffusion Models.

Model	Training (%)	Top-1 Test (%)	Top-5 Test (%)
DiffusionFR	97.55	92.02	95.17
DiffusionFR_noTSD	92.41	89.51	91.96
DiffusionFR_Gaussian	97.20	91.76	93.98

**Table 10:** Accuracies for Data with Different Light Reflection Noise Effects.

Model	Indicator	D_0_E_0_	D_0.6_E_100_	D_0.6_E_250_	D_0.6_E_400_	D_0.8_E_100_	D_0.8_E_250_	D_0.8_E_400_	D_1.0_E_100_	D_1.0_E_250_	D_1.0_E_400_
DiffusionFR	Training (%)	97.55	97.28	97.13	96.91	96.66	96.52	96.28	96.08	95.87	95.74
	Top-1 Test (%)	92.02	91.78	91.58	91.34	91.14	90.95	90.75	90.56	90.37	90.19
	Top-5 Test (%)	95.17	94.90	94.70	94.54	94.31	94.11	93.96	93.70	93.54	93.30
DiffusionFR_VGG16	Training (%)	91.78	91.54	91.33	91.17	90.94	90.72	90.53	90.29	90.16	89.89
	Top-1 Test (%)	86.38	86.13	85.96	85.74	85.49	85.36	85.12	84.94	84.70	84.55
	Top-5 Test (%)	89.48	89.20	89.02	88.82	88.67	88.40	88.20	88.06	87.86	87.64
DiffusionFR_MobileNetv3	Training (%)	93.05	92.82	92.56	92.40	92.18	91.96	91.83	91.63	91.41	91.18
	Top-1 Test (%)	87.55	87.33	87.06	86.90	86.68	86.52	86.30	86.10	85.91	85.74
	Top-5 Test (%)	90.60	90.37	90.15	89.93	89.75	89.56	89.36	89.12	88.93	88.72
DiffusionFR_Tripmix-Net	Training (%)	93.43	93.15	92.97	92.74	92.55	92.41	92.15	91.98	91.76	91.59
	Top-1 Test (%)	88.07	87.81	87.64	87.45	87.25	87.02	86.84	86.58	86.46	86.25
	Top-5 Test (%)	91.21	90.92	90.71	90.52	90.37	90.11	89.92	89.78	89.52	89.35
DiffusionFR_ResNeXt	Training (%)	94.32	94.12	93.89	93.65	93.51	93.27	93.08	92.91	92.63	92.50
	Top-1 Test (%)	88.90	88.69	88.42	88.29	88.07	87.89	87.60	87.49	87.24	87.10
	Top-5 Test (%)	91.98	91.68	91.57	91.38	91.18	90.89	90.76	90.48	90.28	90.13
DiffusionFR_DAMNet	Training (%)	93.80	93.51	93.33	93.18	92.97	92.73	92.60	92.32	92.17	91.90
	Top-1 Test (%)	88.26	88.06	87.86	87.56	87.36	87.26	87.06	86.81	86.62	86.37
	Top-5 Test (%)	91.38	91.14	90.94	90.72	90.51	90.31	90.11	89.91	89.72	89.51
DiffusionFR_ResNet34	Training (%)	96.22	95.98	95.76	95.55	95.36	95.13	94.96	94.76	94.57	94.40
	Top-1 Test (%)	90.80	90.54	90.35	90.17	89.99	89.74	89.59	89.39	89.12	88.95
	Top-5 Test (%)	93.85	93.63	93.44	93.20	93.02	92.81	92.61	92.36	92.24	91.98
DiffusionFR_ResNet101	Training (%)	94.35	94.07	93.86	93.66	93.46	93.33	93.07	92.87	92.66	92.46
	Top-1 Test (%)	88.92	88.65	88.50	88.24	88.04	87.87	87.65	87.48	87.25	87.11
	Top-5 Test (%)	91.72	91.47	91.25	91.11	90.85	90.64	90.49	90.22	90.08	89.89
DiffusionFR_EfficientNet	Training (%)	96.10	95.86	95.61	95.37	95.25	94.94	94.80	94.63	94.43	94.23
	Top-1 Test (%)	90.48	90.43	90.20	89.99	89.87	89.57	89.40	89.26	88.96	88.81
	Top-5 Test (%)	93.38	93.52	93.32	93.03	92.87	92.63	92.48	92.20	92.10	91.87
DiffusionFR_neuro-heuristic	Training (%)	96.82	96.33	96.16	95.93	95.74	95.61	95.35	95.12	94.93	94.75
	Top-1 Test (%)	91.25	90.88	90.58	90.42	90.20	89.99	89.82	89.65	89.39	89.24
	Top-5 Test (%)	94.27	93.98	93.71	93.57	93.31	93.21	93.05	92.77	92.59	92.31
DiffusionFR_BPPD	Training (%)	97.02	96.63	96.48	96.28	96.11	96.01	95.77	95.57	95.40	95.25
	Top-1 Test (%)	91.49	91.40	91.13	90.99	90.80	90.61	90.47	90.32	90.09	89.56
	Top-5 Test (%)	94.63	94.33	94.08	93.97	93.73	93.66	93.52	93.27	93.11	92.86
DiffusionFR_CNN(r1, r2)	Training (%)	97.33	96.89	96.86	96.62	96.41	96.18	96.07	95.76	95.56	95.37
	Top-1 Test (%)	91.69	91.54	91.28	91.01	90.76	90.72	90.40	90.28	90.15	89.83
	Top-5 Test (%)	94.80	94.64	94.33	94.23	93.97	93.88	93.57	93.34	93.28	92.95

**Table 11:** Accuracies for Data with Different Water Ripple Noise Effects.

Model	Indicator	F_0_A_0_	F_0.04_A_2_	F_0.06_A_6_	F_0.08_A_10_
DiffusionFR	Training (%)	97.55	95.45	95.00	94.56
	Top-1 Test (%)	92.02	89.96	89.55	89.10
	Top-5 Test (%)	95.17	93.12	92.75	92.33
DiffusionFR_VGG16	Training (%)	91.78	89.64	89.19	88.93
	Top-1 Test (%)	86.38	84.35	83.94	83.43
	Top-5 Test (%)	89.48	87.32	86.96	86.58
DiffusionFR_MobileNetv3	Training (%)	93.05	91.05	90.54	90.24
	Top-1 Test (%)	87.55	85.44	85.10	84.58
	Top-5 Test (%)	90.60	88.53	88.11	87.77
DiffusionFR_Tripmix-Net	Training (%)	93.43	91.30	90.86	90.57
	Top-1 Test (%)	88.07	85.88	85.49	85.14
	Top-5 Test (%)	91.21	89.19	88.73	88.39
DiffusionFR_ResNeXt	Training (%)	94.32	92.15	91.91	91.38
	Top-1 Test (%)	88.90	86.75	86.36	86.02
	Top-5 Test (%)	91.98	89.90	89.52	89.02
DiffusionFR_DAMNet	Training (%)	93.80	91.76	91.37	90.91
	Top-1 Test (%)	88.26	86.14	85.76	85.39
	Top-5 Test (%)	91.38	89.20	88.82	88.47
DiffusionFR_ResNet34	Training (%)	96.22	94.10	93.79	93.30
	Top-1 Test (%)	90.80	88.62	88.32	87.95
	Top-5 Test (%)	93.85	91.75	91.33	90.88
DiffusionFR_ResNet101	Training (%)	94.35	92.28	91.79	91.54
	Top-1 Test (%)	88.92	86.77	86.51	86.04
	Top-5 Test (%)	91.72	89.71	89.13	88.77
DiffusionFR_EfficientNet	Training (%)	96.10	94.03	93.77	93.21
	Top-1 Test (%)	90.48	88.61	88.27	87.87
	Top-5 Test (%)	93.38	91.72	91.27	90.84
DiffusionFR_neuro-heuristic	Training (%)	96.82	93.61	93.34	92.76
	Top-1 Test (%)	91.25	88.14	87.78	87.36
	Top-5 Test (%)	94.27	91.19	90.72	90.26
DiffusionFR_BPPD	Training (%)	97.02	94.94	94.43	94.07
	Top-1 Test (%)	91.49	89.46	89.02	88.58
	Top-5 Test (%)	94.63	92.58	92.28	91.81
DiffusionFR_CNN(r1, r2)	Training (%)	97.33	95.23	94.71	94.22
	Top-1 Test (%)	91.69	89.64	89.28	88.85
	Top-5 Test (%)	94.80	92.74	92.40	92.10

## Data Availability

The code for our proposed model DiffusionFR and the dataset used in the experiments can be found on GitHub: https://github.com/zafucslab/DiffusionFR (accessed on 30 September 2023).
